# Users and providers collaborate to develop self-management resources

**Published:** 2012-09-04

**Authors:** Gail Butt, Liza McGuinness

**Affiliations:** British Columbia Centre for Disease Control, University of British Columbia, Canada; University of British Columbia, Canada

**Keywords:** chronic illness, self-management, health literacy, collaboration

## Abstract

**Purpose:**

This 2009–11 national project engaged Canadians affected by hepatitis C, a complex chronic illness, and health and social service providers in the development of resources to assist affected individuals learn how to identify and discuss their needs with care providers, navigate complex service systems, and improve their self-care.

**Theory and methods:**

A collaborative and cyclical approach was used to incorporate varied perspectives in the development and implementation of learning materials. Multiple communication modes were used to work effectively with diverse and geographically dispersed stakeholder participants. Stakeholders volunteered their time and made decisions by consensus.

**Results and conclusions:**

A national stakeholder network with 470 participants emerged. Culturally appropriate self-learning resources were created for and with three distinct groups, English, French and Aboriginal. Materials included booklets, posters, and pocket cards available in print and electronic versions including an audio version of key messages. Materials were disseminated through the stakeholder network in multiple ways e.g. stakeholders planned 34 knowledge exchange events where 3366 booklets were distributed. Material’s evaluations were positive and content was considered appropriate to other chronic illnesses.

Uptake of materials surpassed expectations demonstrating that a geographically dispersed network can function effectively to support the learning needs of people living with a complex chronic illness.

